# Experimental evaluation of intra-abdominal adhesions comparing two different intraperitoneal meshes and the effect of a natural anti-inflammatory product on their formation^1^

**DOI:** 10.1590/ACB351205

**Published:** 2021-01-20

**Authors:** Paulo Vicente dos Santos, Rafael Silva Santos, Sydney Correia Leão, Ivisson Xavier Duarte, Sonia Oliveira Lima

**Affiliations:** I Fellow Master degree, Postgraduate Program in Health and Environment, Universidade Tiradentes, Aracaju-SE, Brazil.; II Graduate student, Universidade Tiradentes, Aracaju-SE, Brazil.; III Pathologist, Universidade Tiradentes, Aracaju-SE, Brazil.; IV Pathologist, Universidade Tiradentes, Aracaju-SE, Brazil.; V PhD, Full Professor, Postgraduate Program in Health and Environment, Universidade Tiradentes, Aracaju-SE, Brazil.

**Keywords:** Surgical Mesh, Hernia, Rats

## Abstract

**Purpose:**

In laparoscopic incisional hernia repair, meshes with a tissue-separating barrier are positioned intraperitoneally. Despite this property, the close contact between mesh and viscera involves a risk of adhesion formation. Some natural products, such as red propolis (RP), could reduce these adhesions owing to their anti-inflammatory properties. This study aimed to compare two different intraperitoneal meshes with respect to their characteristics of adhesion formation, histological findings and evaluate the role of RP in the development of these adhesions.

**Methods:**

40 Wistar rats received placement of two different meshes (Symbotex and Dynamesh IPOM) on peritoneum. The animals were divided into two groups: control group (mesh) and treatment group (mesh and RP). After 7 and 14 days, 20 animals of each group underwent midline laparotomy to determine the adhesions and histological characteristics.

**Results:**

Out of the 40 animals, there were two deaths in the test group and two in the control group. All animals in both groups developed adherence to the mesh. At postoperative day (POD) 7, two Symbotex meshes presented firm adhesions and at POD 14, two Dynamesh meshes had firm adhesions as well. The comparison between the meshes under the effect of RP in relation to the control group showed no statistical difference.

**Conclusions:**

Both meshes showed intraperitoneal adhesions in all evaluated samples with similar results on the characteristics of adhesions. RP showed no effect on the incidence or gradation of intraperitoneal adhesions with the mesh.

## Introduction

Incisional hernia is one of the most common postoperative complications, developing in 33% of all surgeries involving peritoneal cavity opening. Approximately 80 - 95% of cases develop between 6 months and 3 years after the initial surgical procedure and can lead to unfavourable aesthetic results and, mainly, loss of quality of life and risk of death due to conditions such as incarceration and strangulation^1^^,^ 2.

In laparoscopy, meshes with a tissue-separating barrier positioned intraperitoneally can cause adhesions, fibrosis, chronic pain and, in more severe cases, entero-enteric fistulas. This is due to a foreign body reaction secondary to the inflammatory response triggered by the presence of this prosthesis inside the peritoneal cavity^3^–^5^. Currently, there are several meshes used for this purpose in the market, but studies on the incidence of adhesions with their use are limited.

Some natural products have anti-inflammatory properties; hence, they can be used in experimental studies for this purpose, including the use of hernia repair mesh^6^^,^ 7. A natural product with the same potential is red propolis, a variant of propolis, produced by bees. The main components of red propolis are flavonoids and phenolic acids, which have anti-inflammatory and antioxidant activities^8^.

This study aimed to compare two different meshes with respect to their characteristics of adhesion formation and integration to the abdominal wall and histological findings and evaluate the role of red propolis in the development of these adhesions.

## Methods

### Ethical aspects

This research project complied with the rules of the National Council for Animal Experimentation and was approved by the Animal Ethics Committee of Tiradentes University (under opinion No 254875).

### Meshes

The Symbotex mesh is a synthetic, nonabsorbable mesh made of three-dimensional polyester (PE) monofilaments coated with bioabsorbable collagen film on its visceral side. Polyester is the result of the reaction of alcohol with carboxylic acid and is a strong, durable and hydrophilic material. It has pores of 2.3 - 3.3 mm and 62 g/m^2^, has multiple filaments and is nonabsorbable. The DynameshIPOM mesh is made of polyvinylidene fluoride (PVDF) monofilaments on its visceral side and polypropylene (PP) on the abdominal side. It is a monofilament with pores > 1 mm and 60 g/m^2^ in the polypropylene portion and 108 g/m^2^ in the PVDF portion. Both meshes were a donation from the companies^9^^,^ 10.

### Obtaining red propolis extract

The red propolis was collected in its raw form from apiaries. Subsequently, the sample was crushed and kept for 1 h in a beaker containing 625 mL of 70% alcohol. After this period, the solution was maintained in an ultrasound bath for 1 h. The extract was filtered and the solvent was eliminated by evaporation in an oven at 45 °C for 48 h, obtaining the dry extract. After drying, the material was weighted and scraped from the plates, forming a dry base of red propolis extract (RPE). The dry extract was suspended in 2% Tween 80 diluted at 10 mg of the extract for each 1 mL of solution (10 mg/mL).The dose used in this study corresponds to 10 mg of the extract for 1 kg of the animals used (10 mg/kg)^8^.

### Experimental design

A total of 40 Wistar rats weighing between 250 and 350 g were used. The animals were housed in the vivarium with a natural light-dark cycle, adjusted environmental temperature and humidity, and feed and water ad libitum. The rats were divided into two groups:

- Control group: 20 rats undergoing midline laparotomy with placement of intraperitoneal PP/PVDF mesh on the right side and PE/collagen mesh on the left side. This group was used to compare the meshes studied and as a control for the group that received the natural product.
- Treatment group: 20 rats undergoing midline laparotomy with placement of intraperitoneal PP/PVDF mesh on the right side and PE/collagen mesh on the left side associated with oral administration of RPE.

Red propolis extract was administered by gavage one day before the first midline laparotomy, constituting a study on the acute effect of this substance and maintained daily during the experiment for the treatment group. For the control group, in the same period, 2% Tween 80 was administered in the same volume.

Postoperatively, the animals received water and feed ad libitum. Seven and 14 days after the surgery (POD 7, POD 14), 20 animals in each group underwent new midline laparotomy in which macroscopic analysis was performed and part of the abdominal wall containing the meshes was removed for histological evaluation.

### Surgical procedure

The animals were anaesthetised with intraperitoneal injection of 50 mg/kg ketamine hydrochloride and 20 mg/kgxylazine hydrochloride.

**Figure 1:**
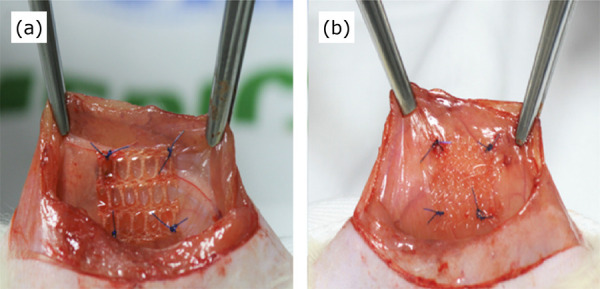
Mesh fragments fixed to the abdominal wall. PE/collagen **(a)** PP/PVDF **(b)**.

After shaving and antisepsis, an 8 cm long incision was made in the ventral part of the abdomen, involving the skin, aponeurosis and peritoneum, reaching the peritoneal cavity. Thereafter, two fragments of meshes measuring 1.5 cm^2^ were interposed and fixed to the abdominal wall 1 cm from the midline, with four simple stiches using 4-0 polypropylene. On the abdominal wall, a PP/PVDF mesh was randomly placed on the right side and a PE/collagen mesh on the left side (Fig. 1). Finally, the abdominal wall was sutured using a 3-0 nylon thread with continuous sutures.

After complete post-anaesthesia recovery, the animals were placed in appropriate cages with a maximum of three animals per cage, being offered water and feed ad libitum until the next surgical procedure.

### Euthanasia of the animals and macroscopic postmortem examination

At 7 and 14 POD, the animals were euthanised using a CO_2_ chamber. Then, necroscopy was performed and the macroscopic aspects related to the mesh were observed. The degree of adhesions between the organs and meshes (adhesion scoring), area of adhesion between the surface of the mesh and intraperitoneal structures (adhesion coverage on the mesh surface), and integration of the mesh into the abdominal wall (tissue integration score) were evaluated^11^–^13^ (Tables 1 to 3).

**Table 1:** Adhesion scoring^13^.

Score		Characteristics
0		Without adhesions
1		Flimsy adhesions: easily removed with blunt dissection and results in limited bleeding
2		Intermediate adhesions: removed with more aggressive blunt dissection or little sharp dissection, results in moderate bleeding and good plane of dissection present
3		Firm adhesions: removed only with sharp dissection, bleeds heavily and no plane of dissection present.

**Table 2:** Assessment of area of adhesion coverage on the mesh surface^11^.

Adhesion		Grade
0%		0
1 - 25%		1
25 - 50%		2
50 - 75%		3
> 75%		4

**Table 3:** Tissue integration score^12^.

Score		Tissue integration
A		Integration of more than 70% of mesh surface
B		Integration of up to 70% of mesh surface area
C		Moderate integration; no tissue ingrowth through perforation holes and less than 50% of mesh surface integrated

### Histological analysis

After the macroscopic analysis, 7 and 14 POD, a 1.6 × 1.6 cm fragment containing all layers of the abdominal wall was collected for histological evaluation.

After fixation, the tissue samples were histologically processed. Six serial histological sections of each block were obtained from each anatomical segment sample and stained using haematoxylin and eosin. The degree of inflammation (lymphocytic infiltration, polymorphonuclear leukocyte infiltration and giant cells) and fibrosis were evaluated semi quantitatively, as negative (-) or positive (+), (++), (+++)^13^.

### Statistical analysis

The variables were described using absolute and relative percentage frequency. The associations between categorical variables were analysed using the Pearson’s chi-square test and estimated using the Monte Carlo method or Fisher’s exact test. The software used was the R Core Team 2018 and p < 0.05.

## Results

Two animals of the treatment group died on POD 11 and 13 and two animals of control group died on POD 12 and 13 due to infection and surgical wound dehiscence. The two meshes used in the experiment were compared with respect to the degree of firmness of the adhesion, percentage of the mesh surface affected by the adhesions, incorporation into the abdominal wall and histopathological findings at 7 and 14 POD (Table 4).

**Table 4:** Results of adhesions, integration and histological findings on 7^th^ and 14^th^ postoperative days.

	Variables evaluated		POD 7				p				POD 14		p
			PP/PVDF n (%)		PE/COLLAGEN n (%)				PP/PVDF n (%)		PE/COLLAGEN n (%)		
Inflammation													
	Negative -		0 (0)		0 (0)		1.000^F^		0 (0)		0 (0)		0.424^F^
	Positive +		4 (40)		4 (40)				4 (50)		8 (100)		
	Positive ++		6 (60)		6 (60)				4 (50)		0 (0)		
Fibrosis													
	Negative -		2 (20)		0 (0)		1.000^QM^		0 (0)		4 (50)		0.208^QM^
	Positive +		2 (20)		4 (40)				4 (50)		4 (50)		
	Positive ++		6 (60)		6 (60)				4 (50)		0 (0)		
Tissue integration													
	A		10 (100)		8 (80)		1.000^F^		6 (75)		4 (50)		1.000^QM^
	B		0 (0)		0 (0)				2 (25)		2 (25)		
	C		0 (0)		2 (20)				0 (0)		2 (25)		
Area of adhesion													
	1-25%		2 (20)		2 (20)		1.000^QM^		0 (0)		6(75)		0.056^QM^
	25-50%		4 (40)		6 (60)				6 (75)		0 (0)		
	50-75%		2 (20)		0 (0)				2 (25)		2 (25)		
	>75%		2 (20)		2 (20)				0 (0)		0 (0)		
Adhesion Scoring													
	Flimsy		4 (40)		2 (20)		1.000^QM^		6 (75)		8 (100)		1.000^F^
	Intermediate		6 (60)		6 (60)				0 (0)		0 (0)		
	Firm		0 (0)		2 (20)				2 (25)		0 (0)		

PE = polyester; POD = postoperative day; PP = polypropylene; PVDF = polyvinylidene fluoride. N = absolute frequency; % = relative percentage frequency. ^QM^ = Pearson Chi-square test estimated via Monte-Carlo procedure. ^F^ = Fisher’s exact test.

QM = Pearson Chi-square test estimated via Monte-Carlo procedure.

F = Fisher’s exact test.

**Figure 2:**
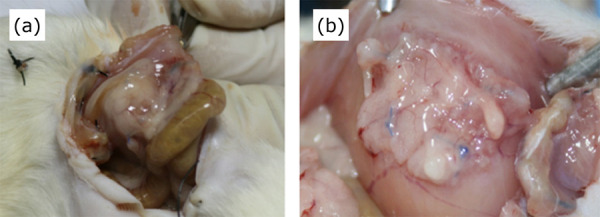
Adhesions between loops and mesh **(a)** and epiploon and mesh **(b)**.

The results showed that both meshes presented adhesions with intraperitoneal structures in all evaluated animals (Fig. 2). The adhesion scoring varied among the studied animals. At POD 7, two PE/collagen meshes presented firm adhesions. The others presented flimsy and intermediate scoring. At POD 14, two PP/PVDF meshes had firm adhesions and all others had flimsy adhesions. In both periods, there was no statistical difference in this aspect between the studied meshes (*p* = 1.000).

As for the assessment of area of adhesion coverage on the mesh surface, at POD 7 two meshes of each type had > 75% adhesion. At POD 14, none of the meshes presented with this classification, being therefore categorised into other classifications. From a statistical point of view, there were no differences in the mesh surface between 7 (*p* = 1,000) and 14 POD (*p* = 0.056).

**Figure 3:**
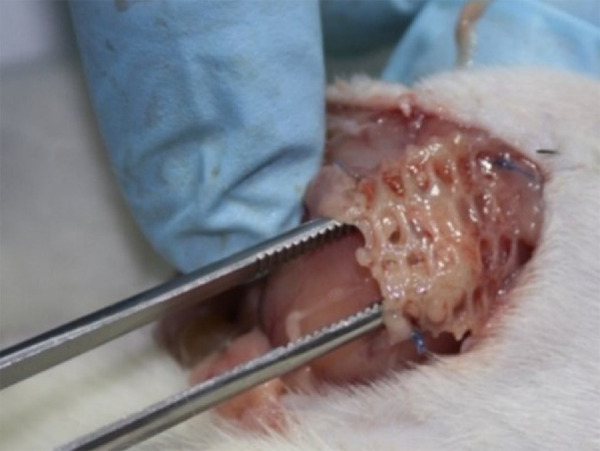
Polyester/collagen mesh with less than 50% of integration.

**Figure 4:**
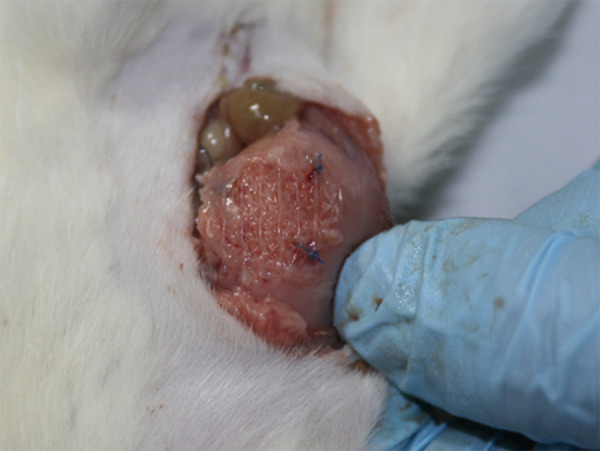
Polypropylene/PVDF mesh with more than 70% of integration (score A).

As for the integration of the mesh into the wall, at POD 7 two PE/collagen meshes presented an incorporation of < 50% and all others an incorporation of > 70%. Moreover, at POD 14 two PE/collagen meshes had an integration of < 50% (Fig. 3) and the other meshes were classified as A and B (Fig. 4). From a statistical point of view, there were no differences in integration between the meshes (*p* = 1,000).

The histological characteristics studied also presented no statistical difference. In both meshes and periods studied, they showed similar results on the presence of fibrosis and severity of inflammation.

The comparison between the two meshes under the effect of the natural product in relation to the control group showed no statistical difference for all criteria evaluated in both periods. In this group, which received RPE, the characteristics of adhesions and histological findings between the evaluated meshes were similar to those in the group that did not receive the natural product (Table 5). 

**Table 5:** Variables evaluated on the 7^th^ and 14^th^ postoperative days (POD) using red propolis extract in relation to the control group.

	Variables evaluated	POD 7											p-value	POD 14										p-value
		PP/PVDF						PE/collagen						PP/PVDF					PE/collagen					
			Test n (%)		Control n (%)		p-value		Test n (%)		Control n (%)				Test n (%)	Control n (%)		p-value		Test n (%)		Control n (%)		
Inflammation																								
	Negative -		2(20)		0 (0)		0.162^QM^		4 (40)		0 (0)		0.462^QM^		0 (0)	0 (0)		0.429^F^		0 (0)		0 (0)		1.000^F^
	Positive +		8 (80)		4(40)				4 (40)		4 (40)				8(100)	4 (50)				6 (75)		8 (100)		
	Positive ++		0 (0)		6 (60)				2 (20)		6 (60)				0 (0)	4 (50)				2 (25)		0 (0)		
Fibrosis																								
	Negative -		0 (0)		2 (20)		1.000^QM^		0 (0)		0 (0)		1.000^F^		2 (25)	0 (0)		1.000^QM^		0 (0)		4 (50)		0.429^F^
	Positive +		2 (20)		2 (20)				2(20)		4 (40)				4 (50)	4 (50)				8(100)		4 (50)		
	Positive ++		8 (80)		6 (60)				8 (80)		6 (60)				2 (25)	4 (50)				0 (0)		0 (0)		
Adhesion Scoring																								
	Flimsy		8(80)		4 (40)		0.524^F^		8 (80)		2 (20)		0.207^QM^		8 (100)	6 (75)		1.000^F^		8(100)		8(100)		*
	Intermediate		2 (20)		6 (60)				2 (20)		6 (60)				0 (0)	0 (0)				0 (0)		0 (0)		
	Firm		0 (0)		0 (0)				0 (0)		2 (20)				0 (0)	2 (25)				0 (0)		0 (0)		
Tissue integration																								
	A		10 (100)		10 (100)		*		8 (80)		8 (80)		1.000^QM^		6 (75)	6 (75)		1.000^F^		4 (50)		4 (50)		1.000^QM^
	B		0 (0)		0 (0)				2 (20)		0 (0)				2 (25)	2 (25)				4 (50)		2 (25)		
	C		0 (0)		0 (0)				0 (0)		2 (20)				0 (0)	0 (0)				0 (0)		2 (25)		
Area of adhesion																								
	1-25%		2(20)		2 (20)		1.000^QM^		4 (40)		2 (20)		1.000^QM^		2 (25)	0 (0)		1.000^QM^		8(100)		6 (75)		1.000^F^
	25-50%		4 (40)		4 (40)				4 (40)		6 (60)				6 (75)	6 (75)				0 (0)		0 (0)		
	50-75%		2 (20)		2 (20)				0 (0)		0 (0)				0 (0)	2 (25)				0 (0)		2 (25)		
	>75%		2 (20)		2 (20)				2 (20)		2 (20)				0 (0)	0 (0)				0 (0)		0 (0)		

PE = polyester; POD = postoperative day; PP = polypropylene; PVDF = polyvinylidene fluoride. N = absolute frequency; % = relative percentage frequency.

QM = Pearson Chi-square test estimated via Monte-Carlo procedure.

F = Fisher’s exact test.

## Discussion

Meshes that could be placed intraperitoneally were developed with the advancement of laparoscopic surgeries. Thus, there would be two benefits: one related to minimally invasive surgery and the other related to the absence of incisional complications. These meshes are different from the classical ones for having a tissue-separating barrier on their visceral face, which was developed to avoid the contact of intraperitoneal structures directly with the mesh and consequently the formation of adhesions and even fistulas. They are also known as double-faced meshes, first used in 1993, which are made of a material in their muscular face that stays in contact with the musculature, inducing an inflammatory reaction capable of maintaining the tensile strength of the tissues and preventing the recurrence of hernia. In the visceral face, which remains in contact with intracavitary structures, they are made of different materials with the same property of preventing these adhesions: polylactic acid, hydrogel, titanium, polyglycolic acid, carboxymethyl cellulose and bovine or porcine collagen. All these materials have the ability to reduce the inflammatory response and consequent fibroplasia, responsible for adhesions between tissues^9^^,^ 14.

The two meshes studied in this experiment have the characteristic of nonadhesions with intraperitoneal structures and, for this reason, are marketed for use in laparoscopic surgeries with intraperitoneal positioning. Although they have this physical characteristic, they are not free of complications related to adhesions and fistulas, two common complications with the use of meshes without this property. Some of these complications are more severe and need urgent repair surgery, whereas others cause nonspecific symptoms and chronic pain. These meshes started to become popular about 20 years ago and their long-term effects are still a cause of uncertainty^5^. In this study, the characteristics of two different brands and models of intraperitoneal meshes available in the market for human use were compared. From a statistical point of view, there were no differences in the variables studied between the meshes but all animals studied showed some type of adherence between intraperitoneal structures and meshes. In four cases, two with each mesh, the adhesions were classified as firm and had loops of the small intestine, large intestine and liver. The clinical interpretation of these findings in humans is worrying, as they could progress with severe complications, including fistulas and faecal peritonitis^4^. The results of studies on humans with this same mesh are conflicting, as a study performed with 344 patients undergoing incisional hernia surgery showed good results with this material^15^, whereas other studies presented important surgical complications with the same mesh, even requiring reoperations in five patients owing to the high incidence of adhesions with intestinal obstruction^5^^,^ 16. Another study also evaluated the PP/PVDF mesh in humans, presenting a risk of reoperation in 6% and chronic pain in 19% of the examined patients^17^. Postoperative ileum was also described as a complication after the use of this mesh^18^. The use of meshes in this position is questioned in the literature regarding safety and there is a tendency to place meshes with no contact with intraperitoneal structures.

Although there was no difference from a statistical point of view, the comparison between the two models studied revealed that the PE/collagen mesh showed a tendency of lower integration with the muscle wall in both periods studied, owing to worse incorporation scores. This incorporation failure with the presence of a space between the mesh and the abdominal wall demands greater care in its fixation because it may trigger the formation of internal hernias.

Experimental studies on the use of meshes without the influence of natural products are common in the literature and evaluate several conditions. The effectiveness of meshes with tissue-separating barriers is the main characteristic evaluated. An experimental study using pigs reported that the PP/PVDF mesh also showed a high incidence of adhesions (83%) when positioned intraperitoneally^19^. A comparison between the different meshes available is also common in the literature, although the two meshes evaluated in this study have not been compared so far^13^^,^ 20 ^,^ 21.

In the present study, RPE was used with the hypothesis that it reduced the presence of adhesions. In the peritoneal cavity, these meshes promote a physiological foreign body reaction, followed by an inflammatory response, depending on the chemical nature and surface area of the mesh in contact with the intracavitary structures, consequently forming adhesions^22^^,^ 23. Some anti-inflammatory substances found in propolis, such as caffeic acid, quercetin, naringenin and caffeic acid phenethyl ester, contribute to the suppression of prostaglandins and leukotrienes synthesis by macrophages and have inhibitory effects on myeloperoxidase activity, NADPH-oxidase, ornithine decarboxylase, tyrosine-protein-kinase and nitric oxide production. Red propolis also contains polyphenols and a wide range of other compounds capable of removing excessive free radicals from the organism. Despite its anti-inflammatory properties, RPE had no effect on adhesion formation. The dose used in this experiment (10 mg/kg) was tested in another study on anti-inflammatory action with doses between 10 and 30 mg/kg^8^. Future experiments in this same line of investigation should test the upper limit of the dose, the use of RPE applied inside peritoneal cavity or even added to the mesh to evaluate if it reduces adhesion formation. This study had some limitations, such as the lack of quantitative and qualitative knowledge of active substances in the extract used for the study that could be done by means of High-performance liquid chromatography.

## CONCLUSIONS

The PE/collagen and PP/PVDF meshes showed similar results on the characteristics of the intraperitoneal adhesions studied and histological changes evaluated. All evaluated samples showed intraperitoneal adhesions involving the omentum, intestinal loops and liver. The natural product evaluated, RPE, despite its anti-inflammatory properties, showed no effect on the incidence or gradation of intraperitoneal adhesions with the mesh.
